# Contingency in Political Philosophy

**DOI:** 10.1007/s11406-016-9802-z

**Published:** 2017-05-10

**Authors:** Susan Mendus

**Affiliations:** Department of Politics, University of York, York, YO10 5DD UK

**Keywords:** Contingency, Liberal moralism, Realism, John Horton, John Rawls

## Abstract

The paper examines John Horton’s realist political theory, in particular his critique of John Rawls’s “high” or “liberal moralism”, and seeks to determine the extent to which, together with Horton, we would have reasons to leave Rawls’s and other Rawlsian accounts behind. The paper argues that some of the insights of Horton’s realism are mistaken, whereas many of those which are not mistaken are compatible with liberal moralism correctly understood. The argument is also formulated in terms of contingency, in particular in terms of a contrast between the realist emphasis on the contingency of human existence and the liberal moralism’s neglect or inability to properly account for it, due to a strong focus on necessity.

In his now famous (if not notorious) Introduction to the first series of *Philosophy, Politics and Society,* Peter Laslett delivered the funeral oration for political philosophy. ‘For the moment, anyway’ he declared ‘political philosophy is dead’. And he went on to note that ‘a survey of our philosophical periodicals … gives the impression that their editors have often included articles on political subjects merely out of a sense of their conventional duty. Their contributors, too, sometimes give the feeling that they have turned their attention to politics only because the curriculum of the university requires it’.^1^ Two years later, in 1958, Robert Dahl echoed Laslett’s sentiments when he wrote ‘In the English-speaking world, where so many of the interesting political problems have been solved (at least superficially), political theory is dead… In the West, this is the age of textual criticism and historical analysis, when the student of political theory makes his way by rediscovering some deservedly obscure text or reinterpreting a familiar one’.^2^ And in similar vein, Brian Barry noted that, in 1961, ‘you could turn over whole volumes of the philosophical journals and find nothing about political philosophy – indeed very little substantive moral philosophy except for an occasional piece of utilitarian casuistry’.^3^ In short, and as we know, in the 1950s and 60s, political philosophy languished in the doldrums and was largely neglected by philosophers and political scientists alike.

However, and as we also know, all this changed in 1971 with the publication of John Rawls’ *A Theory of Justice*. This was the book which, as Thomas Nagel put it, ‘changed the subject’. It was the book which Charles Fried referred to as ‘magisterial’, Marshall Cohen as ‘peerless’, and Stuart Hampshire as ‘the most important work of social and political thought since World War II’. So, even if political philosophy appeared to be dead in the 1950s and 60s, by the 1970s it had risen like a phoenix from the ashes, and its revitalisation was attributable in large part to the liberal theory advanced by John Rawls in his 1971 book. Following the publication of *Theory* it was no longer possible to lament the demise of political philosophy, or to regret the absence of interest in it amongst journal editors. On the contrary, interest in and enthusiasm for Rawlsian liberalism was widespread – even epidemic.

But times have changed yet again, and in the new millennium, liberalism in general and Rawlsian liberalism in particular have been subjected to sustained and sometimes bitter attack. For better or worse, it is no longer possible to read modern political philosophy as a series of footnotes to Rawls, much less is it possible to suppose (as some have done) that, for us, ‘now and around here’, liberalism is the only game in town.^4^ On the contrary, Rawlsian liberalism, which is sometimes referred to as ‘high liberalism’^5^ or ‘liberal moralism’^6^, has been widely criticized, and enthusiasm for it has given way to enthusiasm for a more ‘realist’ approach to political philosophy or political theory.^7^

In this paper, my general aim is to voice some reservations about the strength of the realist attack on Rawlsian liberalism, and my more specific aim is to take issue with the precise form of the attack which is to be found in John Horton’s recent work. In a number of papers, Horton has both distanced himself from high liberalism, or liberal moralism, and allied himself with realism. My aim here is to query the wisdom of those moves, to suggest that some of the alleged insights of realism are mistaken, and to argue that many of the insights which are not mistaken are compatible with liberal moralism properly understood. Most specifically, liberal moralism, at least in its original, 1971, form, is very well able to accommodate Horton’s major criticisms of it, and is certainly able to respond to his central concerns about contingency.

The paper falls into two parts: in the first part, I say something about realism in general and also about the specific form of realism which Horton endorses. To anticipate, I note the extent to which Horton’s account of realism seems (to me, anyway) to focus on the contingent character of our lives, and the extent to which his commitment to realism is motivated by this recognition of contingency and by the conviction that Rawlsian liberalism is either neglectful of it or unable to accommodate it.^8^ Then, in the second part of the paper, I argue, *pace* Horton, that liberal moralism, at least as it appears in Rawls, is just as alert to the contingency of life as is realism. Indeed, it is the importance and ubiquity of contingency that motivates Rawlsian liberalism in the first place. Insofar, then, as Horton’s interest is in the importance of contingency in our lives (and I think that is a large part of his interest) he has no reason to abandon Rawls. Or so I will argue.

## What is Realism?

I begin, however, with the question ‘what is realism?’ Acknowledging that this is a difficult question to answer, William Galston, in an article entitled ‘Realism in Political Theory’ begins by offering a list of ‘charter members’, amongst whom he includes:

*British theorists such as Bernard Williams, Stuart Hampshire, John Dunn, Glen Newey, Richard Bellamy, Geoffrey Hawthorne, Raymond Geuss and John Gray … left Nietzscheans, mainly American, such as William Connolly and Bonnie Honig; Machiavellians such as Chantal Mouffe and (in a different register) Mark Philp … some scholars influenced by Quentin Skinner and the ‘Cambridge historical school’, Judith Shklar and her many admirers* 9

This is a long and diverse list, and it is, as Galston readily acknowledges, very hard to see how this disparate group of people can be united. However, undaunted, he insists that they do in fact ‘share a coherent and formidable dissenting position’ and, as defined by Galston, that position is one which sees high liberalism (ie Rawlsian liberalism) as insufficiently political. Galston writes:

*In this paper realism will emerge as a kind of community stew where everyone throws something different into the pot. There is, however, a theme or sentiment that unites realists at the threshold – the belief that high liberalism represents a desire to evade, displace or escape from politics.*

And it is clear that Horton shares this conviction that ‘high liberalism’ is insufficiently attuned to the political. In an article entitled ‘Realism, Liberal Moralism and a political theory of modus vivendi’ he refers to the realist claim that liberal moralism is descriptively deficient and writes:

*One would get a rather odd idea of what politics is actually like, even in societies that come closest to meeting the conditions of liberal moralism, if one had only liberal moralism as one’s guide … For example, one could read the entire corpus of Rawls’s work without ever having much sense of the seminal role of political parties, pressure groups, and such like. Even elections figure as little more than the right to vote*.^10^

And he concludes with the reflection that ‘*generally, the conception of politics [in liberal moralism] appears etiolated, antiseptic and impossibly high-minded’.* 11

Well, perhaps. I confess, I am not fully persuaded that the absence of detailed discussion of institutions, political parties, and elections, in and of itself, makes Rawls’ theory ‘antiseptic’ or ‘high minded’, but whatever the truth on that, I sense in Horton’s critique of liberal moralism, an additional, and perhaps deeper, concern. Although, like most realists, he laments liberalism’s neglect of politics, his deeper concern (or so it seems to me) is with liberalism’s neglect of contingency, and it is this feature of his position which will be my primary focus in this paper. I turn now, therefore, to explaining and substantiating this claim.

In ‘Realism, Liberal Moralism and a political theory of modus vivendi’ Horton identifies two inter-related complaints which realists make against liberal moralists. The first, which I have already referred to, is that liberal moralism is ‘descriptively deficient’. It ignores politics. The second, however, is that it is ‘normatively irrelevant’ because utopian and practically naive. In spelling out this latter criticism he writes:

*The pull that contrasting, and often conflicting, claims of welfare, need, opportunity and desert (Rawls rejects desert) have for many of us simply do not admit of tidy resolution. Nor is there any good reason to think that they should do. For instance, if there is any truth to value pluralism, it will not necessarily be possible harmoniously to reconcile all the legitimate conflicting values in play in complex societies like our own. Nor is there any reason to think that in practice seeking to remedy one injustice may not create or exacerbate another.* 12

And he goes on to note that:

*What is essential to a modus vivendi is that arrangements are broadly accepted by those subject to them … Crucially, though, what is found acceptable is always a contingent and circumstantial matter, and something that has to be settled by those involved; there is no good reason to think that the appropriate content of any modus vivendi can be determined in advance, for example by political theorists, of the workings of the political process itself … All of this makes a political theory of modus vivendi both richer and messier than liberal moralism.* 13

Drawing on these quotations, and on the general argument of ‘Realism, Liberal Moralism and a political theory of modus vivendi’, it seems to me that much of Horton’s concern about liberal moralism is the concern that it neglects, or under-estimates, the sheer messiness of the real world: the difficulty of reconciling different values harmoniously, the impossibility of solving moral problems without remainder, the futility (maybe even the muddle-headedness) of supposing that the application of rules or principles will serve to solve the difficulties and dilemmas which characterize our lives. It is all this which I gesture towards under the general title ‘contingency’. And it is this emphasis on contingency, rather than an emphasis on political reality, which I take to be the most significant motivating force behind Horton’s turn to realism.

If I am right about this, then it delivers a very distinctive form of realism. Much of the realism which has its inspiration in Machiavelli or in Hobbes has a strongly combative - even aggressive - character. It highlights the importance of power, and emphasises the grubby, and sometimes violent, necessities associated with gaining and retaining power. It is, in this sense, uncompromisingly political and, in general, it is resistant to an understanding of political philosophy as ‘applied moral philosophy’ or to any assertion of the priority of the moral over the political.^14^

By contrast, forms of realism which are rooted in a recognition of contingency owe their fundamental insights to moral philosophy. They draw upon the work of anti-utilitarians such as Bernard Williams, or those particularists who take their inspiration from the work of the later Wittgenstein. Both groups construe morality as something beyond the mere application of moral rules or principles, and both emphasise the importance of context in analysing and assessing moral life. More generally, both groups deny that morality can be reduced to rules, and argue instead for a recognition of the sheer messiness of the moral world. To gain something of the flavour of these writers’ thinking, consider the following passage from D Z Phillips’ article ‘Some Limits to Moral Endeavour’:

*My complaint in this essay has been that [the moral theories of Hare, Foot and Melden] have attempted to be altogether too tidy and all-embracing … they present a picture of ordered moral priorities and optimism. There is little indication of the ‘social machinery which jolts us round and along’. I have sought to correct this picture by providing reminders of some limits to moral endeavour. It may well be true that where paying attention to moral considerations is concerned, the reapers are relatively few, but it should not be assumed that a ready-made harvest awaits those who attempt to reap, that success inevitably crowns the endeavours of men of good will.* 15

And a similar line of thinking may be discerned in the work of Bernard Williams, both in his criticisms of utilitarianism, and in his discussions of ancient Greek thought, where he notes:

*That the world was not made for us, or we for the world, that our history tells no purposive story, and that there is no position outside the world or outside history from which we might hope to authenticate our activities. We have to acknowledge the hideous costs of many human achievements that we value, including this reflective sense itself, and recognise that there is no redemptive Hegelian story or universal Leibnizian cost-benefit analysis to show that all will come out well in the end.* 16

Both Phillips and Williams are concerned to emphasise the messiness of moral life, the difficulty of reconciling all values harmoniously, and the impossibility, despite all our best efforts, of guaranteeing moral success in our endeavours.

If I am right in thinking that Horton’s realism springs, in large part, from his concern with contingency of this kind, then several thoughts follow – thoughts both about the way in which his own realist theory might be developed, and about who his real opponents are. As I have already indicated. I am not persuaded that the Rawlsian liberals either are or should be the real targets of his attack and so, in an attempt to fulfil my earlier promise, I now identify a few ways in which Rawlsian liberalism can in fact respond to the problems identified by what I refer to as ‘contingent realism’.

There are three strands to my argument here: in the first sub-section I suggest that Rawlsian liberalism can in fact respond to the contingency of life and indeed it is largely motivated by recognition of the contingency of life. In the second sub-section I raise some questions about the role and significance of the contingent in politics (as distinct from morality), and in the third and final sub-section I offer a limited defence of moralism in politics. As foreshadowed earlier in the paper, my overall aim is to defend liberal moralism (Rawlsian liberalism) against the attack from contingent realists.

## The Significance of Contingency

### Contingency in Rawls

First, then, the role of the contingent in Rawlsian liberalism. Just to recall, in his discussion of the alleged normative irrelevance of liberal moralism, Horton writes:

*The pull that contrasting, and often conflicting, claims of welfare, need, opportunity and desert (Rawls rejects desert) have for many of us simply do not admit of tidy resolution. Nor is there any good reason to think that they should do. For instance, if there is any truth to value pluralism, it will not necessarily be possible harmoniously to reconcile all the legitimate conflicting values in play in complex societies like our own. Nor is there any reason to think that in practice seeking to remedy one injustice may not create or exacerbate another.* 17

As I read it, there are two facets to Horton’s concern here, and the first is that Rawlsian liberalism is insufficiently attentive to the persistence and permanence of pluralities of values. Now, it is well known that, at least in his later writings, Rawls insists both on the permanence of pluralism and indeed on the inappropriateness of regretting the permanence of pluralism. So it might be said that this part of Horton’s concern is ill-founded. Rawls not only recognises that values are plural, the recognition that they are plural, and are likely to remain so for the foreseeable future, is his starting point. Nonetheless, many writers share Horton’s concern about liberal commitment to pluralism. In particular, many have pointed to the fact that, for Rawls, pluralism extends only over conceptions of the good, not over conceptions of right. And this is, to put it mildly, puzzling. Thus, Jeremy Waldron writes:

*Pluralism of comprehensive religious, philosophical and moral doctrines is not the only pluralism with which we have to deal in a modern democratic society. We also have to deal with justice-pluralism and disagreement about rights. Maybe political philosophy should be required to come to terms with that circumstance also.* 18

Maybe it should, and of course much of Waldron’s own brand of realism (if such it is) takes the form of an argument to the effect that it should. I won’t engage in that dispute here, but simply note that, even if the restriction of pluralism to conceptions of the good stands in need of justification, it does not follow that liberal moralists like Rawls are utterly unaware of pluralism. It does not follow that they think (as Horton implies) that it will be possible harmoniously to reconcile all the legitimate conflicting values in play in complex societies like our own. It may be true that Rawls does not make enough space for the plurality of values, but it is quite untrue to say that he makes none at all.^19^

Similarly, Rawls’ recognition of the contingency of life is very well-documented. Indeed the argument for the two principles of justice, and especially for the difference principle, rests crucially on the claim that skills and talents are arbitrary from a moral point of view. Of course, it may be said that a recognition of arbitrariness is different from a recognition of contingency, but it seems to me that the two concepts are very close to one another and, at root, both are concerned with what Horton refers to as the ‘messiness’ of ordinary life – the extent to which things are outside our control, the difficulties associated with finding clean and final answers to moral and political problems.

### Contingency in Political Philosophy

What, then, of the role of the contingent in politics (as distinct from its role in life generally and in moral life in particular)? I have suggested that Horton’s enthusiasm for realism or, perhaps better, his reservations about liberal moralism, are traceable to the importance he attaches to contingency, and I have also noted the centrality of the contingent in the writings of anti-utilitarians such as Bernard Williams, and Wittgensteinian particularists such as D Z Phillips. I referred to, and quoted from, D Z Phillips’ ‘Limits of Moral Endeavour’ which is a plea for the ‘messiness’ of moral life and a reminder of the difficulty (perhaps impossibility) of resolving moral problems ‘without remainder’. In that same article Phillips speaks of the gap between moral theory and moral reality (much as the realists speak of the gap between political theory and political reality), and he says, in tones which are reminiscent of Horton’s thoughts about the shortcomings of liberal moralism:

*When we turn from these tidy philosophical accounts* [the accounts offered by Hare, Foot and Melden] *of the ways in which moral beliefs place limits on human actions to look at actual situations, do we not want to accuse these accounts of an over simplification and falsification of the facts?.* 20

I think my own answer is ‘Yes’, I do want to accuse them of that. However, I am far from sure that accusing them (the moral theorists) of that should prompt me to endorse realism. Recognition of contingency has, or may have, very different implications for political theory, on the one hand, and moral theory, on the other. Or so it seems to me. I cannot defend this thought fully here, but it depends in some part on the conviction that although life is indeed complex, messy, arbitrary, shot through with contingencies which cannot be eradicated, and replete with problems which cannot be solved without remainder, nonetheless one very important role of politics is precisely to attempt, as far as possible, to mitigate the worst effects of those contingencies. In *A Theory of Justice* Rawls famously notes the arbitrariness of the distribution of natural talents, when he writes:

*But it does not follow that one should eliminate these distinctions. There is another way to deal with them. The basic structure can be arranged so that these contingencies work for the good of the least fortunate. Thus we are led to the difference principle if we wish to set up the social system so that no-one gains or loses from his arbitrary place in the distribution of natural assets or his initial position in society without giving or receiving compensating advantages in return.* 21

Put differently, Rawls fully acknowledges the contingency of life, but insists that politics is the forum in and through which those contingencies can, to some extent, be ‘tamed’. The ‘social system’ can (and should) be so organised as to mitigate the worst effects of arbitrariness. Of course, it may not be possible to eliminate them entirely, and it may indeed be naively utopian to imagine that one can. On the other hand, however, to refrain from using the mechanisms of the state in pursuit of this end is surely indefensible. Bernard Williams makes something like this point in *Shame and Necessity* when he writes:

*Even if we cannot, and perhaps should not, cancel all effects of mere necessity and luck, at least we hope that they can be placed within a framework that raises the question of justice and can answer it in such a way that the necessities will not be radically coercive and the luck will be no worse than luck.* 22

Williams’ final point is crucial: even if we cannot eradicate all luck, all contingency, all arbitrariness, we can try to ensure that ‘the luck is no worse than luck’. We can try to ensure that inequalities, for instance, are not the result of the exercise of untrammelled and unjustified power. And it is surely one of the main aims of politics to do just that. Politics is precisely the area in which arbitrary inequalities can be ‘smoothed, and luck can be (to some extent) compensated for.

So, again, if we give prominence to the contingent, as I think Horton does, then the distance between ‘contingent’ realism and liberal moralism (Rawlsian liberalism) may be less great than is often supposed. Rawlsian liberalism can indeed acknowledge the permanence of contingency, and its aim, as a form of liberal moralism, is to show how the apparatus of the state may be used to minimize that contingency.

However, it may be thought that even this is too much – too moralistic, too high-minded, too naively utopian. So let me turn, finally and briefly, to a limited defence of liberal moralism against the realist attack, and in doing this, I take my cue from an article on multiculturalism by Duncan Ivison.

### Contingency and Realism

In an article entitled ‘The Moralism of Multiculturalism’ Ivison defends liberal multiculturalists against the charge of undue moralism. His focus is on the plight of Australian Aborigines (specifically on the Stolen Generation), and his concern is with the allegation that the multiculturalist policies proposed in this case are essentially continuous with the racist and colonial policies they are meant to replace. He cites the concerns of journalists and politicians that ‘since it is power, not moral argument, that shapes social and political interaction, moral argument without a transformation of the relations of power is a form of vacuous moralising.^23^

The allegation here, in the specific case of Aboriginal claims, echoes the more general allegation made by realists (I refer here to Galstonian realists rather than contingent realists) against liberal moralists. That allegation, to recall, is that liberal moralism is neglectful of and naive about the realities of power in political life. In this specific case the allegation is that liberal multiculturalists threaten to do more harm than good when they cling to their abstract moral principles in disregard of the consequences of applying those (ideal) principles in a very non-ideal world. So, as the editorial in *The Australian* newspaper put it:

*Ideals, indulged without any sense of realism, can obscure and do great damage. The most painful example of this is the reconciliation crusade, a cause that [has] effectively blinded the purists to the crisis of violence and sexual abuse unfolding across Aboriginal Australia. Purism, though, is far more appealing in its essence, than a cool pragmatic appraisal of the landscape.* 24

Indeed it is. Or can be. It is, I think, undeniable that cleaving to principle in disregard of practicalities has done tremendous damage in the Aboriginal case, and it is hard to believe that it is the only case where that charge can legitimately be levelled.^25^ But it does not follow, and it is not true, that the best response is realism. This is partly because, and as Ivison is at pains to point out, politics is never entirely about power. Power, he notes, ‘always seeks to legitimise itself in some way, or at least to delegitimate its opponents, and thus always leaves itself open to the counter-legitimizing moves and arguments of others. Insofar as politics is constantly dealing with questions of legitimacy, morality is in some way intrinsic to it.^26^

And there, of course, is the rub. Whatever the realists may say, politics is never entirely about power, but always about the legitimate exercise of power. But insofar as realists concede that politics is about legitimate power, they risk sliding into the very moralism which they themselves reject. This is not to say that the slide must be either complete or inevitable, but it is a slide which is potentially damaging to the realist project, as that is usually understood ie as it is understood by many of the writers identified by Galston.^27^ To put the point provocatively, the realist insistence on the ubiquity of power in politics needs to be set against the recognition that, in reality – that is to say in the case of real conflicts in the real world – it is not, or not usually, power which is invoked, but legitimate power. Insofar as realists ignore this, they are less realistic than they ought to be.

## Conclusion

The aim of this paper has been to offer a limited defence of liberal moralism against the criticisms offered by various kinds of realist. My central suggestion has been that the form of realism which Horton seems to endorse is one which gives centrality to contingency - to the messiness of life, the arbitrariness of fate, and the irreconcilability of diverse values. This form of realism, I have argued, is very different from a realism that is grounded primarily in Hobbesian considerations about the ubiquity of power or Machiavellian considerations about the practicalities of securing political success. It is a form of realism which is compatible with Rawlsian liberalism, and indeed Rawls’ own liberalism is motivated in no small part by his recognition of the importance of contingency in our lives. Therefore, insofar as Horton is troubled by the contingency of life, he has no occasion to abandon liberal moralism and quite a few reasons for cleaving to it.

## Footnotes

1 Peter Laslett, Introduction to *Philosophy, Politics and Society*, eds. Peter Laslett and W.G. Ruciman, Oxford, Blackwell, 1956, p. vii.

2 Robert Dahl ‘Political Theory: Truth and Consequences’ in *World Politics,* Vol.11, No.1, October 1958, pp. 89-102.

3 Brian Barry ‘The Strange Death of Political Philosophy’, in *Democracy and Power,* Oxford, Clarendon, 1991, pp. 11-23.

4 For a critique of this line of thinking see the title essay in Bernard Williams, *In the Beginning Was the Deed: Realism and Moralism in Political Argument,* Princeton, University Press, 2005, pp.18-28.

5 This term is used by Galston in ‘Realism in Political Theory’, *European Journal of Political Theory*, Vol. 9(4), 2010, pp. 385-411.

6 ‘Liberal moralism’ is John Horton’s favoured term.

7 In this paper, I use the terms ‘political philosophy’ and ‘political theory’ interchangeably.

8 The precise meaning of ‘contingency’ will, I hope, become clear in the next section.

9 Op.cit., p.386.

10 John Horton ‘Realism, Liberal Moralism and a Political Theory of Modus Vivendi’ in *European Journal of Political Theory,* Vol. 9 (4), 2010, p. 433

11 Ibid. This quotation again highlights the extent to which the realist challenge is made in terms which are to put it mildly, loaded.

12 Op.cit., p.436.

13 Op.cit, pp.439–440.

14 For more on this, see Bernard Williams ‘Realism and Moralism in Political Theory’ in *In the Beginning was the Deed,* op.cit., pp. 1-17.

15 D Z Phillips ‘Some Limits to Moral Endeavour’ in *Through a Darkening Glass,* Oxford, Wiley-Blackwell, 1982, p.46, p.49.

16
*Shame and Necessity,* Oxford, University of California Press, 1993, p. 166.

17 Op. cit.

18 Jeremy Waldron ‘Rawls’s Political Liberalism’ in Waldron *Law and Disagreement,* Oxford, Clarendon, 1999, pp.158-9.

19 For a defence of Rawls on this, see Thomas Nagel ‘Rawls and Liberalism’ in Samuel Freeman (ed) *The Cambridge Companion to Rawls,* Cambridge, University Press, 2003, pp. 62-85.

20 Op.cit, p. 37.

21
*A Theory of Justice,* Oxford, University Press, section 17.

22
*Shame and Necessity,* op.cit., p.128. See also p.164.

23 D. Ivison ‘The Moralism of Multiculturalism’ *Journal of Applied Philosophy,* Vol 22, No 2, 2005, p. 174.

24 As quoted in Ivison, op,cit,, p. 172.

25 See, for example, some of the cases cited in Brian Barry, *Culture and Equality,* Oxford, Polity, 2001.

26 Op. Cit,., p. 177.

27 Horton mentions this danger in the article I am focusing on and notes that Bernard Williams' theory, and especially his BLD (Basic Legitimation Demand), risks sliding into this problem.

